# Anesthésie Locorégionale dans un Pays à Ressources Limitées: La Lidocaïne 1,5% Adrenalinée, Alternative à la Ropivacaine 0,5% Pour le Bloc Axillaire Échoguidé

**DOI:** 10.48327/EFX9-0P51

**Published:** 2021-01-29

**Authors:** J. Donamou, M.L. Bah, A. Bangoura

**Affiliations:** 1 Service d'anesthésie-réanimation de l'Hôpital national Ignace Deen, BP 1042 Conakry, Guinée; 2 Service de traumatologie de l'Hôpital national Ignace Deen, BP 1042 Conakry, Guinée

**Keywords:** Lidocaïne, Ropivacaïne, Anesthésie, Efficacité, Coût, Chirurgie, Hôpital, Conakry, Guinée, Afrique sub-saharienne, Lidocaine, Ropivacaine, Anesthesia, Effectiveness, Cost, Surgery, Hospital, Conakry, Guinea, Sub-Saharan Africa

## Abstract

**Objectif:**

L'objectif de l'étude était d'évaluer l'administration de la lidocaïne 1,5% adrénalinée comme alternative à la ropivacaïne 0,5% pour le bloc axillaire échoguidé.

**Méthode:**

Il s'agissait d'une étude prospective et randomisée d'une durée de 28 mois (du 15 janvier 2017 au 15 mai 2019) réalisée dans le service d'anesthésie de l'Hôpital national Ignace Deen de Conakry (Guinée).

**Résultats:**

Au total, 38 patients ont été recrutés: 19 patients dans chaque groupe. Leur âge moyen était de 46 ± 17 ans dans le groupe lidocaïne adrénalinée contre 44 ± 20 ans dans le groupe ropivacaïne. Le délai moyen d'installation du bloc dans le groupe lidocaïne adrénalinée était de 6,8 ± 2,1 minutes contre 8,3 ± 2,4 minutes dans le groupe la ropivacaïne (p = 0,04). La durée d'action moyenne était de 233 ± 57 minutes dans le groupe lidocaïne adrénalinée contre 260 ± 74 minutes dans le groupe ropivacaïne (p= 0,21). L'efficacité du bloc était identique dans les 2 groupes avec 89,5% des blocs efficaces dans le groupe lidocaïne adrénalinée et dans le groupe ropivacaïne (p =1). Le coût des consommables pour le groupe lidocaïne adrénalinée était de 15 euros versus 60 euros pour le groupe ropivacaïne.

**Conclusion:**

La lidocaïne 1,5% adrénalinée est une bonne alternative à la ropivacaïne 0,5% pour la réalisation des blocs axillaires échoguidés dans les pays à ressources limitées.

## Abstract (en)

**Objective:**

The aim of this study was to evaluate lidocaine 1.5% adrenaline as an alternative to ropivacaine 0.5% for the echo guided axillary block.

**Methode:**

We conducted a 28-month prospective and randomized study (January 15, 2017 to May 15, 2019) conducted in the anesthesia department of the Ignace Deen National Hospital in Conakry, Guinea.

**Results:**

A total of 38 patients were enrolled: 19 in each group. Their average age was 46 ± 17 years in the lidocaine group compared to 44 ± 20 years in the ropivacaine group. The average time to fix the block in the lidocaine group was 6.8 ± 2.1 minutes compared to 8.3 ± 2.4 minutes in the ropivacaine group (p = 0.04). The average duration of action was 233 ± 57 minutes in the lidocaine group versus 260 ± 74 minutes in the ropivacaine group (p= 0.21). The block efficiency rate was identical in both groups with 89.5% of effective blocks in the lidocaine group and in the ropivacaine group (p =1). The cost of consumables for the lidocaine group was 15 euros compared to 60 euros for the ropivacaine group.

**Conclusion:**

1.5% adrenaline lidocaine is a good alternative to ropivacaine 0.5% for the production of echoguided axillary blocks in resource-limited countries.

## Introduction

Le bloc axillaire échoguidé est un bloc d'utilisation courante dans la chirurgie du membre supérieur [6]. Plusieurs anesthésiques locaux peuvent être utilisés pour obtenir un bloc efficace, en particulier la ropivacaïne et la lidocaïne (Xylocaïne™) adrénalinée.

En pratique clinique, ces anesthésiques locaux diffèrent par leurs propriétés pharmacologiques et pharmacodynamiques [4]. La ropivacaïne est un anesthésique local de grande puissance anesthésique. Elle se caractérise par un délai d'installation court, une durée d'action longue et une faible neurotoxicité et cardiotoxicité [1, 5]. La lidocaïne quant à elle, est un anesthésique local de puissance moyenne. Elle a une durée d'action qui varie en fonction du site d'injection et de l'adjonction ou non d'un vasoconstricteur [1, 5]. Son délai d'installation est court et dépend de la concentration de la solution anesthésique [1]. Ces 2 anesthésiques locaux sont fréquemment utilisés pour l'anesthésie locorégionale [4, 6]. Cependant, ils n'ont jamais fait l'objet d'une comparaison dans le cadre du bloc axillaire échoguidé.

La présente étude avait pour objectif d'évaluer la lidocaïne 1,5% adrénalinée comme alternative à la ropivacaïne 0,5% pour le bloc axillaire échoguidé.

## Patients et Méthodes

Il s'agissait d'une étude prospective et randomisée en simple aveugle, réalisée sur une période de 28 mois (du 15 janvier 2017 au 15 mai 2019) dans le service d'anesthésie de l'Hôpital national Ignace Deen de Conakry.

Après avoir obtenu l'approbation du comité d'éthique pour la recherche en santé (N°084/CNERS/19) et le consentement éclairé de tous les patients, nous avons inclus dans cette étude 38 patients ASA I et II, âgés de 12 à 80 ans devant avoir un bloc axillaire échoguidé pour une ostéosynthèse des 2 os de l'avant-bras. Nous avons exclu toutes les ostéosynthèses de l'avant-bras réalisées sous anesthésie générale: les autres critères d'exclusion étaient une allergie suspectée ou connue aux anesthésiques locaux et un refus du patient.

Les malades ont été répartis dans les 2 groupes: 19 patients dans le groupe lidocaïne adrénalinée et les 19 autres dans le groupe ropivacaïne. La nature de l'anesthésique local injecté était déterminée par randomisation à partir d'une liste aléatoire proposant le protocole du groupe lidocaïne ou du groupe ropivacaïne. Les anesthésiques locaux ont été fournis gratuitement dans les 2 groupes.

Les blocs axillaires échoguidés ont été réalisés par le même médecin anesthésiste-réanimateur. Tous les patients ont bénéficié d'une consultation pré-anesthésique, 24 heures avant l'intervention. Ils ont été admis au bloc opératoire à jeun, avec une voie veineuse périphérique et prémédiqués par de l'hydroxyzine dichlorhydrate (Atarax™) à la posologie de 1 mg/kg. Les patients ont bénéficié d'une surveillance de la pression artérielle, de la SpO2, et de la fréquence cardiaque. Après le lavage chirurgical des mains de l'opérateur, port des gants stériles et installation du patient, on procédait à l'asepsie large de la zone de ponction et à l'application de gel sur le site d'application de la sonde d'échographie (échographe SonoSite Nano Maxx^®^). Tous les blocs ont été réalisés dans le plan avec une aiguille B. BRAUN, Stimuplex^®^ Ultra 0,7 × 50 mm 22G et une sonde d'échographie linéaire de 12-15 Hz.

Après avoir procédé au repérage échographique des nerfs, l'aiguille était introduite latéralement à la sonde dans le plan et on procédait à son échoguidage jusqu'au niveau des nerfs à anesthésier. Une fois à ce niveau, on injectait un volume bien défini de la solution d'anesthésique locale (lidocaïne 1,5% adrénalinée à 1/200000 ou ropivacaïne 0,5%). Les patients n'avaient pas reçu d'information sur le type d'anesthésique local utilisé pour leur bloc. Pour la lidocaïne 1,5% adrénalinée 1/200000, on injectait un volume total de 30 ml. Ce volume a été déterminé à partir du volume minimum effectif de lidocaïne 1,5% adrénalinée 1/200000 chez 90% des patients pour un bloc axillaire échoguidé [3]. Il était réparti comme suit: 8 ml pour le nerf médian, 8 ml pour le nerf ulnaire, 8 ml pour le nerf radial et 6 ml pour le nerf musculocutané.

Pour la ropivacaïne 0,5%, on injectait un volume total de 23 ml répartis comme suit: 6 ml pour le nerf médian, 6 ml pour le nerf ulnaire, 6 ml pour le nerf radial et 5 ml pour le nerf musculocutané.

Dans les deux groupes, on procédait à l'infiltration sous-cutanée de l'anesthésique local correspondant (3 ml) pour l'anesthésie du nerf cutané médial du bras et de l'avant-bras. L'installation du bloc était évaluée par un test au froid réalisé dans les territoires couverts par les différents nerfs. Le bloc était noté « parfait » ou « efficace » si tous les territoires nerveux correspondant étaient anesthésiés; il était dit « partiel », nécessitant un complément d'anesthésie par une sédation avec du diazépam 5 mg et 100 microgrammes de fentanyl par voie intraveineuse lorsque certains territoires nerveux n'étaient pas parfaitement anesthésiés; il était noté « échec », nécessitant une conversion en anesthésie générale lorsque le bloc n'était pas installé.

L'analgésie postopératoire était assurée chez tous les patients avec du paracétamol codéiné et de l'ibuprofène par voie orale.

Le coût des consommables évalué à la pharmacie était pour le groupe ropivacaïne de 60 euros versus 15 euros pour le groupe lidocaïne.

La satisfaction des patients était appréciée à travers un score de satisfaction propre au service qui était côté de 0 à 10 et réparti en 4 items: score 10 = très satisfait; score 7-9 = satisfait, score 5-6 = moyennement satisfait; score <5 = insatisfait.

Les données ont été collectées sur une fiche d'enquête préétablie comportant les questionnaires qui étaient remplis en préopératoire, en peropératoire et en postopératoire. Pour chaque patient, les paramètres évalués ont été les suivants: le délai d'installation, la durée d'action du bloc, l'efficacité du bloc, la durée de l'intervention, la satisfaction des patients. Le délai d'installation était défini comme le délai nécessaire à l'installation du bloc sensitif après avoir injecté l'anesthésique local sur tous les nerfs. La durée d'action du bloc était le temps écoulé depuis l'installation du bloc sensitif jusqu'à la levée de celui-ci: elle a été évaluée en post-opératoire au cours de cette étude.

Les données qualitatives ont été décrites sous forme de proportions et les données quantitatives sous forme de moyenne ± écart type et de médiane avec des intervalles interquartiles. Selon les cas, le test du Khi-deux ou un test exact de Fisher ont été utilisés pour la comparaison des proportions. Une valeur de p<0,05 a été considérée comme significative. Les données ont été analysées à l'aide du logiciel SPSS version 21 (SPSS Inc, Chicago, Illinois).

## Résultats

L'âge moyen des patients du groupe lidocaïne adrénalinée était de 46 ± 17 ans et de 44 ± 20 ans dans le groupe ropivacaïne (p = 0,77). Le sexe masculin était prédominant dans les 2 groupes avec un sex-ratio de 5,3 dans le groupe lidocaïne adrénalinée et de 3,7 dans le groupe ropivacaïne (p = 0,6). La majorité des patients dans les 2 groupes était ASA I (p = 0,66). La durée moyenne de réalisation du bloc était de 7,5 ± 1,9 minutes pour le groupe lidocaïne adrénalinée et de 8,7 ± 2,1 minutes dans le groupe ropivacaïne (P = 0,07). Le délai moyen d'installation du bloc était de 6,8 ± 2,0 minutes dans le groupe lidocaïne adrénalinée contre 8,3 ± 2,4 minutes dans le groupe la ropivacaïne (p = 0,04). La durée d'action moyenne du bloc était de 233 ± 58 minutes dans le groupe lidocaïne adrénalinée et de 260 ± 75 minutes dans le groupe ropivacaïne (p= 0,21). La durée moyenne d'intervention était de 73 ± 10 minutes dans le groupe lidocaïne adrénalinée et de 85 ± 51 minutes dans le groupe ropivacaïne (p = 0,34). L'efficacité du bloc était identique dans les 2 groupes avec 89,5% des blocs efficaces dans le groupe lidocaïne adrénalinée et dans le groupe ropivacaïne (p =1), 4 patients au total ont eu une sédation complémentaire et aucun patient n'a bénéficié d'une anesthésie générale. La majorité des patients dans les 2 groupes étaient très satisfaits avec des taux de 73,7% pour le groupe lidocaïne adrénalinée et 94,7% pour le groupe ropivacaïne (p = 0,55).

## Discussion

Notre étude a permis de montrer que la lidocaïne 1,5% adrénalinée à 1/200000 peut être une bonne alternative à la ropivacaïne 0,5% pour la réalisation des blocs axillaires échoguidés dans les pays à ressources limitées. Nous avons retrouvé une efficacité de la lidocaïne 1,5% adrénalinée identique à celle de la ropivacaïne. En effet, aucune différence statistique n'a été observée entre les deux anesthésiques locaux concernant l'efficacité du bloc (p = 0,66). Cependant sur le plan pharmacologique, la ropivacaïne 0,5% à une puissance anesthésique supérieure à celle de la lidocaïne 1,5% adrénalinée [1, 5], ce qui fait qu'elle offre un bloc de meilleure qualité que celui de la lidocaïne 1,5% adrénalinée.

Notre conclusion se rapporte au fait que nous n'avons pas cherché à savoir précisément lequel de ces deux anesthésiques locaux était le plus puissant ou le meilleur, mais plutôt, si l'intervention chirurgicale était réalisable sans besoin de recourir à une anesthésie générale ou à une sédation complémentaire; à ce niveau, nous avons observé que la grande majorité des interventions chirurgicales dans les deux groupes ont été réalisées dans les meilleures conditions quel que soit l'anesthésique local utilisé. En effet, la majorité des patients dans les deux groupes ont été très satisfaits. Leur satisfaction peut s'expliquer par la bonne qualité de l'anesthésie dans les deux groupes due non seulement à l'efficacité du bloc, mais aussi à l'utilisation de l'échographie. Cette dernière a permis de visionner les structures nerveuses, les variations anatomiques et la diffusion de l'anesthésique local, améliorant ainsi le taux de succès du bloc. Par ailleurs, l'observation des blocs partiels a quelque peu altéré la satisfaction de certains patients dans les deux groupes sans différence significative. Ces blocs partiels observés dans notre étude étaient dus à un défaut d'identification des nerfs à l'échographie ou à la mauvaise diffusion de l'anesthésique local, plutôt qu'à la nature de l'anesthésique local utilisé. Notre étude nous a aussi permis de noter quelques différences pharmacologiques entre ces deux anesthésiques locaux, notamment au niveau du délai d'installation et de la durée d'action du bloc. Ces données pharmacologiques sont importantes à considérer en pratique clinique dans le choix de l'anesthésique local.

Concernant le délai d'installation, nous avons observé que la lidocaïne 1,5% adrénalinée s'installait plus rapidement que la ropivacaïne. Nos résultats trouvent leur explication dans la pharmacologie de ces anesthésiques locaux. En effet, l'installation du bloc dans un nerf isolé dépend des propriétés physico-chimiques de l'anesthésique local et notamment de son pKa (qui détermine le délai d'action). Ainsi, un anesthésique local tel que la lidocaïne ayant un pKa proche du pH ambiant aura un rapport forme ionisée/forme non ionisée plus faible comparé à un anesthésique local avec un pKa plus élevé tel que la ropivacaïne. Or, c'est la forme non ionisée qui diffuse le plus facilement à travers les membranes des cellules nerveuses. La dose et la concentration de l'anesthésique local interviennent également in vivo. Ainsi, à dose standard de 2%, le délai d'installation de la lidocaïne est de 2 à 3 minutes. Bien entendu, ce délai d'action dépend également du diamètre du nerf à bloquer et donc du site d'injection [7]. Pour ce qui était de la durée d'action moyenne du bloc, elle était plus longue dans le groupe ropivacaïne en comparaison au groupe lidocaïne adrénalinée (260 contre 233 minutes). En effet, la lidocaïne non adrénalinée est un anesthésique local de durée d'action moyenne (90 - 120 minutes), l'adjonction d'un adjuvant comme l'adrénaline (vasoconstricteur) permet de contourner le problème de la durée de bloc en la prolongeant jusqu'à au moins 180 minutes [1, 2, 5, 8].

Le coût des consommables d'anesthésie est une variable importante dans les pays à ressources limitées, car ce sont les patients eux-mêmes qui achètent ces consommables pour leur intervention après la prescription du médecin anesthésiste. Leur coût élevé est un facteur retardant très souvent la prise en charge des patients. L'anesthésiste dans sa prescription doit donc faire un compromis entre l'efficacité du produit anesthésique et son coût. Nous avons constaté que le coût des consommables dans le groupe ropivacaïne était 4 fois plus élevé que celui du groupe lidocaïne adrenalinée. Ce facteur coût non négligeable associé à l'efficacité dont fait preuve la lidocaïne 1,5% adrénalinée pour la réalisation des blocs axillaires échoguidés fait de celle-ci une très bonne alternative à la ropivacaïne 0,5%.

La principale faiblesse de notre étude est son manque de puissance due à la petite taille de l'échantillon. Cependant, les résultats obtenus par ce travail prospectif apportent des informations précises qui n'auraient pu être retrouvées en rétrospectif.

## Conclusion

Notre étude a permis de montrer que la ropivacaïne 0,5% et la lidocaïne 1,5% adrénalinée sont des anesthésiques locaux qui ont des effets comparables en termes d'efficacité et de fiabilité dans la réalisation du bloc axillaire échoguidé. Cependant le surcoût induit par la prescription de la ropivacaïne joue en sa défaveur, car elle coûte 4 fois plus cher que la lidocaïne 1,5% adrénalinée. L'utilisation de cette dernière pourrait alors être une bonne alternative à la ropivacaïne 0,5% pour la réalisation du bloc axillaire échoguidé dans les pays à ressources limitées.

## Conflits D'intérêts

Les auteurs ne déclarent aucun conflit d'intérêts.
